# Integrated care competencies and their association with cross‐cultural competence among registered nurses: A cross‐sectional questionnaire survey

**DOI:** 10.1002/nop2.2062

**Published:** 2023-12-15

**Authors:** Maliheh Nekouei Marvi Langari, Jaana Lindström, Tarja Heponiemi, Anu‐Marja Kaihlanen, Laura Hietapakka, Hamid Heidarian Miri, Hannele Turunen

**Affiliations:** ^1^ Department of Nursing Science University of Eastern Finland Kuopio Finland; ^2^ Population Health Unit, Department of Public Health and Welfare Finnish Institute for Health and Welfare Helsinki Finland; ^3^ Finnish Institute for Health and Welfare Helsinki Finland; ^4^ Health Research Institute University of Limerick Ireland; ^5^ Kuopio University Hospital Kuopio Finland

**Keywords:** cultural competency, delivery of healthcare, immigrant, integrated, nurse

## Abstract

**Aim:**

To examine the association between the integrated care competencies and cross‐cultural competence of registered nurses prior to the integration of social and healthcare services in Finland.

**Design:**

A descriptive correlational cross‐sectional questionnaire survey was conducted.

**Methods:**

A simple random sample of 10,000 registered nurses was drawn from the Finnish Central Register of Valvira (National Supervisory Authority for Welfare and Health); 7000 of them were sent the online questionnaire, and a total of 1232 registered nurses participated in the study. We collected data using background questions, revised versions of the Competent Workforce for the Future tool in the four domains of client orientation, responsibility for personal or relative's welfare, fluency and clarity of services and access to the services and of the Cross‐Cultural Competence of Healthcare Professional tool in the four domains of motivation/curiosity, attitude, skill and emotion/empathy.

**Results:**

Participants demonstrated a high level of integrated care competencies (mean = 4.00, SD ± 0.49). An association was observed between integrated care competencies and their domains of skills, motivation/curiosity, emotions/empathy, and cross‐cultural competence (*p* < 0.001). Female sex, older age, more working experience, employment in the private sector, and higher self‐rated competence for working in a multicultural environment were positively associated with higher integrated care competencies.

**Conclusion:**

It is recommended that nurse managers and nurse educators emphasize the development of registered nurses' cross‐cultural competence alongside integrated care competencies to meet the needs of different individuals and communities when providing integrated care.

**Patient or Public Contribution:**

Finnish registered nurses including all types of nurses, midwives and paramedics working the public and private healthcare, were involved in this study by responding to the online survey.

## 1 INTRODUCTION

With the increasing incidence of chronic diseases, the increasing population of older adults and the complexity of care for recipients with various health and social needs, integrated care has become one of the leading models for developing healthcare services globally (World Health Organization, 2015a; Zonneveld et al., 2018). The World Health Organization (2015a) introduced people‐centred integrated care and health services as global strategies and described integrated care as lifelong care that encompasses primary, secondary, tertiary and palliative care based on clients' needs. Therefore, people‐centred health systems emphasize the needs of individuals and communities with a participatory nature and decision‐making to address equity and provide high‐quality care (World Health Organization, 2015b). However, the concept of integrated care remains vague (Goodwin, 2016; Zonneveld et al., 2018).

Integrated care, in principle, is comprehensive, equitable, sustainable, coordinated, holistic, empowering, respectful (dignity and cultural values), co‐produced (in collaboration with individuals and communities), evidence‐based and ethical (World Health Organization, 2015a, 2015b; Zonneveld et al., 2018). Succinctly, integrated health service delivery is defined as ‘an approach to strengthen people‐centred health systems through the promotion of comprehensive delivery of quality services across the life‐course, designed according to the multidimensional needs of the population and the individual and delivered by a coordinated multidisciplinary team of providers working across settings and levels of care’ (World Health Organization, 2016, p. 10). Reducing inequalities and improving public health and people‐centred healthcare systems were indicated in the European Policy Framework of Health, 2020 (World Health Organization, 2012) and renewed as the EU Roadmap for the year 2030 (World Health Organization, 2017). However, a gap exists in research on the competencies, training and development of the social care and healthcare workforce in providing integrated care (Barraclough et al., 2021).

## 2 BACKGROUND

### 2.1 Immigrants' health and integrated care

International migration has been increasing globally (McAuliffe & Triandafyllidou, 2021). The European continent received the second‐highest number (22 million) of international migrants from 2000 to 2017 (United Nations, Department of Economic and Social Affairs, 2017). Although the coronavirus disease 2019 (COVID‐19) pandemic has temporarily decreased the immigration rate, in 2021, the proportions of asylum seekers (25%) and illegal Schengen border crossing (56%) increased compared to the previous year (International Center for Migration Policy Development, 2022). In 2020, approximately 1.9 million non‐Europeans immigrated to Europe (Eurostat, 2020). Although infectious diseases are more prevalent among immigrants due to mobility, incomplete vaccination programs, poor health conditions and limited access to healthcare at the beginning of migration, the risk of developing non‐communicable diseases, such as cardiovascular diseases and cancer, increases as immigrants spend a long time in the host country (World Health Organization, 2019). Moreover, immigrants face problems using healthcare services, and the healthcare systems in different European countries experience difficulties in providing them with these services. This is a challenge, particularly in some European countries, due to language differences, insurance coverage, cultural differences, different perceptions of diseases, lack of information about healthcare in the host country and the negative attitudes of healthcare providers (Priebe et al., 2011), and immigrants have reported experiencing racism when using healthcare services (Kang et al., 2019).

Integrated care is an approach to reducing inequalities and disparities by providing high‐quality, comprehensive and efficient healthcare (Davis et al., 2015; Holden et al., 2014). Reorienting the care model is a strategy in the application of integrated and people‐centred care to ensure the effectiveness of healthcare services. Adopting a holistic approach and emphasizing primary healthcare services and the co‐production of health services, integrated care emphasizes health promotion and prevention actions for health and well‐being through specific consideration of care recipients' cultural preferences (World Health Organization, 2016).

Healthcare professionals need to have shared values (Valentijn et al., 2013; Zonneveld et al., 2018), competencies (Nummela et al., 2019) and clinical skills for better communication and further collaboration in providing integrated care. Social care and healthcare professionals need to understand clients comprehensively—their individual perceptions, beliefs, needs and relationships with families—and provide client‐centred care (Barraclough et al., 2021). Essential integrated care competencies (ICCs) emerging from the scoping review of Barraclough et al. (2021) encompasses enhancing workforce understanding across the health and social care systems, developing a deeper relationship with patients and their families, patient‐centeredness, health promotion, disease prevention and interprofessional education and teamwork’ (p. 4).

Cross‐cultural competence (CCC) is a common concept used in health sciences; it extends beyond knowing just one culture by highlighting communication skills, cultural awareness and understanding of health beliefs in general (Epner & Baile, 2012). CCC is an ongoing process and is defined as ‘the ability to work and communicate effectively and appropriately with people from culturally different backgrounds’ (Alizadeh & Chavan, 2016, p. 120). Furthermore, CCC of healthcare professionals includes dimensions of motivation/curiosity, attitudes, skills, knowledge/awareness and emotions/empathy (Bernhard et al., 2015).

Finland, with a population of 5.5 million in northern Europe, hosts 444,031 immigrants with foreign backgrounds, constituting 8.7% of the total population (Statistics Finland, 2020). It has been undertaking historical reformation in health and social services, referred to as *Sote‐uudistus*, and is moving towards integrating health, social and rescue services in 2023. A total of 21 well‐being service counties and hospital districts of Helsinki and Uusima are establishing primary and tertiary healthcare, social and rescue services, replacing 195 health and social services organizations in each municipality and 22 rescue departments (Ministry of Social Affairs and Health, 2023). In integrated health and social services, nurses and social workers need competencies to work efficiently with both native and immigrant populations and understand their health and cultural needs. Nurses, in the frontline of healthcare, confront healthcare reformation. Moreover, their workplace well‐being and stress are associated with the level of integration of services and care (Longpré et al., 2014).

### 2.2 Finnish nursing education and core competencies

Nursing education and qualification vary across countries (Rafferty et al., 2019). In Finland, nurses study for 3.5 years, paramedics and public health nurses for 4 years and midwives for 4.5 years in universities of applied sciences and the National Supervisory Authority for Welfare and Health (Valvira) grants them The licence to work as registered nurses (RNs) (Eriksson et al., 2015). Nurses and paramedics get a practice licence and register as nurses, while public health nurses get a practice licence and register as both nurses and public health nurses. Midwives get a licence and register as midwives and nurses (Finnish Nurses Association, n.d.).

The content of Finnish nursing education follows the European Union Directive 2005/36/EC on the recognition of professional qualifications and regulations (European Union, 2013), but Finnish universities of applied sciences are permitted by law to customize their nursing curricula (Ministry of Education and Culture, 2014). Despite their variation, these curricula contain a series of core competencies, including (1) client‐centredness, (2) ethics and professionality, (3) leadership skills, (4) clinical nursing, (5) evidence‐based practice and decision‐making, (6) education and teaching competence, (7) health promotion, (8) social care and healthcare environment and (9) quality and safety of social care and healthcare services (Eriksson et al., 2015). Although their nursing curricula, workplace and responsibilities vary, RNs are required to demonstrate the above nine core competencies at work. Knowledge of the competencies of the social care and healthcare workforce in providing integrated care and of the association between ICCs and CCC among healthcare professionals, including nurses, is limited.

## 3 AIM

This study aimed to (1) describe integrated care competencies and the cross‐cultural competence of RNs prior to the integration of social and healthcare in Finland, (2) examine the associations of the integrated care competencies and their different domains with cross‐cultural competence and (3) identify the background factors associated with integrated care competencies and cross‐cultural competence.

### 3.1 Design

A descriptive correlational cross‐sectional questionnaire survey was conducted.

### 3.2 Participants

The study participants were Finnish RNs, including nurses, public health nurses, midwives and paramedics.

### 3.3 Instruments validity and reliability

The questionnaire survey included background variables (numerical, nominal and categorical) and the revised versions of two validated tools with categorical items to evaluate the ICCs and CCC. Background variables were the following: sex; age; country of birth; nursing credentials; other degrees; graduation year; manager status; work experience; employment sector (municipality, state, private, etc.); multicultural education as part of the nursing degree; self‐rated competence for working in a multicultural environment (5‐point Likert scale); additional training related to multiculturalism; frequency of having clients from different cultures (5‐point Likert scale) and having a work colleague with a foreign background.

The competent workforce for the future (COPE) tool with 21 items for measuring ICCs has been assessed for construct validity and internal consistency (total Cronbach's *α* = 0.95) in the Finnish population (Nummela et al., 2019). The ICCs were measured in this study using a revised version of COPE tool with 14 items (total Cronbach's *α* = 0.90) rated on a 5‐point Likert scale (1 = *poorly*, 5 = *very well*) that reflected the same four main competencies required for integrated care in the COPE tool: client orientation (four items, e.g. plan the goal of the treatment or service in collaboration with the client); responsibility for personal or relative's welfare (three items, e.g. involve the client's loved ones in planning and implementing the treatment or service); access to services (two items, e.g. explore alternatives or compulsory services together with the client) and fluency and clarity of services (five items, e.g. referring the client to the care path or service chain). Scores ranged from 14 to 70, total mean scores ranged from 1 to 5 and higher score depicted higher ICCs. ICCs were classified into three categories based on the total mean score: low (mean = 1–2), fair (mean = 3) and high (mean = 4–5).

To evaluate CCC, we used a revised Finnish version of the cross‐cultural competence of healthcare professionals (CCCHP) tool originally developed by Bernhard et al. (2015).

The tool has been validated in the Finnish context by Hietapakka et al. (2019). In their study, the CCCHP tool was translated from English to Finnish language by a professional translator, and then back‐translated to English by a native translator proficient in both English and Finnish to ensure the quality of the translation. The Finnish version of the CCCHP tool was tested for structural validity and internal consistency. The result of exploratory and confirmatory factor analysis showed the best fit of the model in four out of five domains of the original tool. These four domains were motivation/curiosity, attitude, skills and emotions/empathy. The internal consistency was reported acceptable with Cronbach's alpha of the four domains ranging between 0.79 and 0.86 (Hietapakka et al., 2019).

In the revised Finnish version of the CCCHP tool, as validated by Hietapakka et al. (2019), we included 13 items from the four domains, selecting those with the highest factor loading (≥0.6). The internal consistency of the tool used in our study was found satisfactory for these four domains with Cronbach's alpha ranging between 0.75 and 0.82. These domains and their number of items in the used tool were motivation/curiosity (three items, e.g. I find it inspiring to work with clients/patients with an immigrant background), attitudes (four items, e.g. I find it stressful that people who moved to Finland a long time ago do not speak Finnish properly), skills (three items, e.g. I take into account the patient's family values, religion, etc. if they appear to be significant for the treatment) and emotions/empathy (three items, e.g. I get frustrated if a patient with an immigrant background does not understand what I am saying). The items were rated on a 5‐point Likert scale (1 = completely disagree, 5 = completely agree). Scores ranged from 13 to 65, total mean scores ranged from 1 to 5 and higher score depicted higher CCC. According to the total mean score, CCC was categorized into three levels: low (mean = 1–2), fair (mean = 3) and high (mean = 4–5).

### 3.4 Sampling and recruitment

First, a simple random sample of 10,000 RNs was picked from the Finnish Central Register of Valvira. Then, we asked the Union of Health and Social Care Professionals in Finland (Tehy) to forward the electronic questionnaire to those RNs by email. However, the Tehy only had the email addresses of 7000 out of the 10,000 RNs who were union members. Finally, the Tehy forwarded the electronic questionnaire to the individual email addresses of 7000 RNs who were members of the union. The questionnaire included the study's aim, study information, the voluntary and anonymous nature of participation and a link to the online survey in Finnish. Three reminders were sent to the nurses during the data collection period between November and December 2018, and a total of 1232 RNs participated in the study (response rate 18%). The power of the statistical test for detecting the association between ICCs and CCC using linear regression model and calculated by PASS, exceeded 99.9%. This calculation was based on the observed coefficient of 0.35, with the standard deviation of ICCs and CCC set at 0.5 and 0.3, respectively, and the alpha level of 5%.

### 3.5 Exclusion criteria

Practical nurses were not included in the study sample as they have degrees from vocational colleges and are not licensed to practice as RNs.

### 3.6 Data analyses

We used Stata version 17 to conduct data analysis and used descriptive statistics (frequencies, percentages, means and standard deviations) to present the participants' general characteristics and their ICCs and CCC; the associations between ICCs, CCC and their dimensions were explored using linear regression. The level of significance was set to 0.05, and the confidence interval was calculated at 95% with one degree of freedom. Regression analysis was also used to explore the association of ICCs and CCC with different background factors presented in Table 1, including continual (i.e. graduation year), binary (i.e. sex) and categorical variables (i.e. employment sector) (Bzovsky et al., 2022). The regression analysis was performed in two steps. First, each background variable was examined using ICCs and CCC in the univariable model. Second, if the *p*‐value of the background variables was less than 0.2 (Bursac et al., 2008), they were entered into the multivariable model. In the final multivariable model for ICCs, the background variables included sex, age, having any other degree, year of graduation, manager status, work experience, employment sector, self‐rated competence for working in a multicultural environment and additional training related to multiculturalism. In the final multivariable model for CCC, the background variables included sex, age, country of birth, having any other degree, self‐rated competence for working in a multicultural environment, additional training related to multiculturalism and having a colleague with a foreign background at work.

## 4 RESULTS

### 4.1 Demographic information of participants

Table 1 depicts the general characteristics of the 1232 RNs who participated in the survey. The majority of participants were women (93%) born in Finland, and nearly half of them were 35–54 years old. Most (76%) of the RNs were nurses and paramedics, while 24% were public health nurses and midwives. One‐third of the participants reported having another degree. Many respondents had worked as nurses for less than 5 years, and the majority worked for the municipality's health services. Nearly half of the participants reported having received multicultural education as part of their nursing degree, and 74% confirmed not receiving any additional training regarding multiculturalism.

**TABLE 1:** General characteristics of the study sample (*n* = 1232).

Variables (*n*)	*N*	%
Gender (1223)		
Man	88	7
Woman	1134	93
Other	1	0.1
Age (years) (1213)		
≤34	262	22
35–54	633	52
≥55	318	26
Country of birth (1222)		
Finland	1197	98
Countries inside and outside the EU/EEA	25	2
Registered nurses (1232)		
Nurses and paramedics	933	76
Public health nurses and midwifes	299	24
Having any other degree (1201)		
Yes	410	34
No	791	66
Graduation year (1185)		
2011–2018	445	38
2010–2001	289	24
≤2000	451	38
Manager status (1218)		
Yes	121	10
No	959	79
Acting like (i.e. charge nurse, team leader)	138	11
Work experience (1189)		
≤5	522	44
6–10	200	17
11–15	133	11
≥16	334	28
Employment sector (1208)		
Municipality	978	81
State	21	2
Private	170	14
Foundation or association	39	3
Having multicultural education in the degree (1225)		
No	542	44
Yes	683	56
Self‐rated competence for working in a multicultural environment (1225)		
Poorly	136	11
Fairly poorly	355	29
Neither poorly not well	461	38
Fairly well	230	19
Very well	43	3
Additional training related to multiculturalism (1226)		
No	913	74
Yes	313	26
Frequency of taking care of clients from different cultures (1224)		
Very rarely or never	134	11
Quite rarely	338	27
Not rarely or often	167	14
Quite often	381	31
Very often or continuously	204	17
Having a foreign background colleague at work (1222)		
No	485	40
Yes	737	60

Abbreviation: EU/EEA, Europe/European Economic Area.

### 4.2 Integrated care competencies and cross‐cultural competence of RNs

The total mean ICCs score for RNs was high (mean = 4.00, SD ± 0.49) in Table 2, which was similar for subgroups of nurses and paramedics (mean = 4.00, SD ± 0.50) and public health nurses and midwives (mean = 4.02, SD ± 0.45). Overall, RNs had higher competence in the client orientation approach (mean = 4.21, SD ± 0.51), enabling clients to make health‐related decisions for themselves and close relatives (mean = 3.99, SD ± 0.59), providing their clients with a clear path in service usage (mean = 3.90, SD ± 0.58) and providing accessibility to different services (mean = 3.85, SD ± 0.71).

The total mean CCC score for RNs was 3.27 (SD ± 0.32), which was similar for subgroups of nurses and paramedics (mean = 3.27, SD ± 0.34) and public health nurses and midwives (mean = 3.28, SD ± 0.29). Generally, RNs reported higher CCC skills (mean = 3.89, SD ± 0.69), motivation/curiosity (mean = 3.78, SD ± 0.79), attitudes (mean = 3.31, SD ± 0.74) and emotions/empathy (mean = 2.09, SD ± 0.80).

**TABLE 2:** Integrated care competencies (ICCs), cross‐cultural competence (CCC) and their dimensions for registered nurses (*n* = 1232).

	Nurses and paramedics (*N* = 933)	Public health nurses and midwives (*N* = 299)	Total registered nurses (*N* = 1232)
	Mean (±SD)	Mean (±SD)	Mean (±SD)
ICCs^a^	4.00 (0.50)	4.02 (0.45)	4.00 (0.49)
Client orientation	4.21 (0.51)	4.21 (0.49)	4.21 (0.51)
Responsibility for personal or relative's welfare	4.00 (0.61)	3.97 (0.55)	3.99 (0.59)
Fluency and clarity of services	3.88 (0.60)	3.95 (0.53)	3.90 (0.58)
Access to the services	3.84 (0.72)	3.89 (0.68)	3.85 (0.71)
CCC^b^	3.27 (0.34)	3.28 (0.29)	3.27 (0.32)
Skills	3.88 (0.69)	3.93 (0.67)	3.89 (0.69)
Motivation/curiosity	3.75 (0.81)	3.87 (0.69)	3.78 (0.79)
Attitudes	3.33 (0.74)	3.26 (0.72)	3.31 (0.74)
Emotions/empathy	2.10 (0.81)	2.05 (0.75)	2.09 (0.80)

^a^ 5‐point Likert scale (poorly = 1, very well = 5).

^b^ 5‐point Likert scale (completely disagree = 1, completely agree = 5).

### 4.3 Association of RNs' integrated care competencies with cross‐cultural competence

As shown in Table 3, RNs' ICCs were associated with CCC (B = 0.35, 95% CI = 0.272–0.435) and all its dimensions, including skill, motivation/curiosity and emotions/empathy (*p* < 0.001), except for attitudes.

Moreover, three out of four dimensions of ICCs (providing accessibility to different services, enabling clients to make health‐related decisions for themselves and close relatives, and client orientation) were associated with CCC and its dimensions, including skill, motivation/curiosity and emotions/empathy (*p* < 0.001), except for attitudes. Only ICCs dimensions—fluency and clarity of services—were associated with all four CCC dimensions.

Negative associations were observed between ICCs and all their dimensions with CCC‐emotion/empathy (feelings, reactions and comfort towards cultural diversity).

**TABLE 3:** Pairwise correlation of integrated care competencies (ICCs) and cross‐cultural competence (CCC) and their dimensions using linear regression analysis (registered nurses *n* = 1232).

Variables		*B*	95% CI	*p*‐Value
ICCs	CCC	0.35	0.272 to 0.435	<0.001
ICCs	CCC‐skills	0.27	0.235 to 0.309	<0.001
ICCs	CCC‐motivation/curiosity	0.15	0.116 to 0.183	<0.001
ICCs	CCC‐attitudes	0.03	−0.006 to −0.067	0.105
ICCs	CCC‐emotions/empathy	−0.12	−0.157 to −0.090	<0.001
ICCs‐access to the services	CCC	0.40	0.285 to 0.524	<0.001
ICCs‐access to the services	CCC‐skills	0.35	0.298 to 0.406	<0.001
ICCs‐access to the services	CCC‐motivation/curiosity	0.18	0.135 to 0.235	<0.001
ICCs‐access to the services	CCC‐attitudes	0.00	−0.046 to 0.061	0.782
ICCs‐access to the services	CCC‐emotions/empathy	−0.15	−0.203 to −0.104	<0.001
ICCs‐fluency and clarity of services	CCC	0.36	0.268 to 0.464	<0.001
ICCs‐ Fluency and clarity of services	CCC‐skills	0.25	0.204 to 0.295	<0.001
ICCs‐fluency and clarity of services	CCC‐motivation/curiosity	0.13	0.092 to 0.174	<0.001
ICCs‐fluency and clarity of services	CCC‐attitudes	0.05	0.013 to 0.102	0.010
ICCs‐fluency and clarity of services	CCC‐emotions/empathy	−0.11	−0.152 to −0.072	<0.001
ICCs‐responsibility for personal or relative's welfare	CCC	0.35	0.256 to 0.458	<0.001
ICCs‐responsibility for personal or relative's welfare	CCC‐skills	0.27	0.232 to 0.324	<0.001
ICCs‐responsibility for personal or relative's welfare	CCC‐motivation/curiosity	0.15	0.115 to 0.199	<0.001
ICC‐responsibility for personal or relative's welfare	CCC‐attitudes	0.01	−0.030 to 0.060	0.516
ICCs‐responsibility for personal or relative's welfare	CCC‐emotions/empathy	−0.11	−0.158 to −0.075	<0.001
ICCs‐client orientation	CCC	0.31	0.227 to 0.398	<0.001
ICC‐client orientation	CCC‐skills	0.25	0.214 to 0.292	<0.001
ICCs‐client orientation	CCC‐motivation/curiosity	0.14	0.113 to 0.184	<0.001
ICCs‐client orientation	CCC‐attitudes	0.02	−0.018 to 0.059	0.298
ICCs‐client orientation	CCC‐emotions/empathy	−0.12	−0.162 to −0.092	<0.001

### 4.4 Association of background factors with integrated care competencies and cross‐cultural competence

In the first regression model (Table 4), women aged 35 years or older, working for 1 year or more in private healthcare, reporting higher competence for working in a multicultural environment and receiving additional training related to multiculturalism had a positive association with higher ICCs (*p* < 0.05).

**TABLE 4:** Association of registered nurses' background variables with integrated care competencies (ICCs) using linear regression analysis.

Independent variable (ref)	Univariable model			Multivariable model		
	*B*	95% CI	*p*‐Value	*B*	95% CI	*p*‐Value
Gender (men)	0.19	0.085 to 0.297	<0.001	0.12	0.015 to 0.231	0.025
Age (≤34 years)						
35–54 years	0.13	0.059 to 0.200	<0.001	0.09	0.005 to 0.174	0.037
≥55 years	0.17	0.099 to 0.259	<0.001	0.12	0.014 to 0.236	0.027
Having any other degree (no)	0.11	0.058 to 0.175	<0.001	0.05	−0.009 to 0.119	0.097
Graduation year	0.00	−0.000 to 0.004	0.094	−0.00	−0.005 to 0.001	0.317
Management position (yes)	−0.04	−0.106 to 0.012	0.125	−0.02	−0.082 to 0.040	0.501
Work experience (<1 year)						
1–2 years	0.13	0.030 to 0.231	0.011	0.12	0.025 to 0.223	0.014
3–5 years	0.13	0.035 to 0.242	0.009	0.11	0.006 to 0.213	0.037
6–10 years	0.14	0.047 to 0.242	0.004	0.13	0.035 to 0.236	0.008
11–15 years	0.19	0.082 to 0.299	0.001	0.15	0.041 to 0.274	0.008
Over 15	0.15	0.070 to 0.245	<0.001	0.15	0.052 to 0.265	0.004
Employment sector (municipality)						
State	−0.02	−0.234 to 0.185	0.819	−0.02	−0.236 to 0.195	0.853
Private	0.08	0.005 to 0.163	0.037	0.08	0.006 to 0.169	0.035
Foundation or association	0.14	−0.011 to 0.298	0.070	0.14	−0.010 to 0.302	0.067
Self‐rated competence for working in a multicultural environment^a^ (poorly)	0.08	0.055 to 0.109	<0.001	0.07	0.042 to 0.100	<0.001
Additional training related to multiculturalism (no)	0.18	0.124 to 0.249	<0.001	0.12	0.061 to 0.194	<0.001

^a^ 5‐point Likert scale (poorly =1 and 5 = very well).

In the second regression model (Table 5), CCC was only associated with sex, and women had higher CCC than men (*p* < 0.001).

**TABLE 5:** Association of registered nurses' background variables with cross‐cultural competence (CCC) using linear regression analysis.

Independent variables (ref)	Univariable model			Multivariable model		
	B	95% CI	*p*‐Value	*B*	95% CI	*p*‐Value
Gender (men)	0.13	0.061 to 0.202	<0.001	0.13	0.063 to 0.208	<0.001
Age (≤34 years)						
35–54 years	0.02	−0.018 to 0.076	0.232	0.02	−0.029 to 0.070	0.416
≥55 years	0.05	−0.003 to 0.104	0.067	0.03	−0.025 to 0.089	0.271
Country of birth (countries inside and outside EU/EEA)	0.09	−0.000 to 0.197	0.050	0.09	−0.008 to 0.194	0.071
Having any other degree (no)	0.04	0.000 to 0.079	0.045	0.01	−0.023 to 0.060	0.378
Self‐rated competence for working in a multicultural environment^a^ (poorly)	0.01	0.000 to 0.037	0.047	0.01	−0.006 to 0.033	0.185
Taking additional training related to multiculturalism (no)	0.06	0.024 to 0.109	0.002	0.04	−0.001 to 0.089	0.056
Having foreign background colleagues at work (no)	0.02	−0.011 to 0.064	0.165	0.01	−0.020 to 0.057	0.348

Abbreviation: EU/EEA, Europe/European Economic Area.

^a^ 5‐point Likert scale (poorly = 1 and 5 = very well).

## 5 DISCUSSION

The study result indicated that RNs had high ICCs. A former study conducted by Flinkman et al. (2016) demonstrated that high quality of care is associated with higher nurse competence. Moreover, the lack of required competencies for nurses affects their stress and distress levels and work well‐being (Kaihlanen et al., 2021). According to the World Health Organization (2015a), integrated care benefits individuals (by increasing satisfaction, access and shared decision‐making with professionals), communities (by improving accessibility for marginalized groups, health‐seeking behaviours, community awareness and trust in care services), healthcare professionals (by enhanced job satisfaction, higher workload, skills and more educational opportunities) and healthcare systems. A strategy for integrated care is community empowerment, with a special focus on minority communities such as immigrants (World Health Organization, 2015b). Empowering people or communities means providing support to involve them in healthcare and to take active roles in co‐producing health services and making effective health‐related decisions (World Health Organization, 2015b). However, the shortage of nurses has proved a global challenge (Nekouei Marvi Langari et al., 2020), and turnover intention among nurses has increased during the COVID‐19 pandemic (Tolksdorf et al., 2022). Likewise, the Ministry of Economic Affairs and Employment of Finland (2022) reported a shortage of nurses and social workers compared to pre‐COVID‐19. Therefore, implementing integrated care in the country may alleviate emotional exhaustion and burnout among nurses through collaborative care and a sense of high‐level personal accomplishment by fulfilling patients' needs (Zubatsky et al., 2020). The paucity of research on the competencies of professionals for the integrated care and related training highlighted the emerging global need for developing degree curricula, models to support education and competencies for integrated care (Barraclough et al., 2021).

In our study, among the four dimensions of ICCs, RNs had the highest competence in client orientation. This finding aligns with a study by Lahtinen et al. (2022), which indicated that increasing nurses' competence leads to higher patient‐centred care competence. Having a client‐oriented mindset and facilitating client engagement in the treatment plan are client orientation skills and reflect competence in providing client‐centred care (Nummela et al., 2019), which is one of the nurses' core competencies in the Finnish nursing curricula (Eriksson et al., 2015) and in integrated care (Barraclough et al., 2021; Nummela et al., 2019). ‘Client‐centred care’, ‘patient‐centred care’ and ‘person‐centred care’ are interchangeable terms used in health sciences (McMillan et al., 2013). Patient‐centred care emphasizes partnership with individuals to consider their culture and belief in health services (Jo Delaney, 2018) and rejects the paternalist model of care where doctors know everything and make the diagnosis; rather, it provides a more holistic approach and focuses not only on the disease and pathology but also on the individual experiencing the illness (Bogaert et al., 2022). This approach is used to redesign healthcare to enhance health outcomes and client satisfaction (Jo Delaney, 2018). Patient‐centred care can improve satisfaction, well‐being and self‐management among clients (Rathert et al., 2013) and is closely related to cultural competence (Epner & Baile, 2012). Therefore, healthcare professionals are required to possess high CCC to provide effective client‐centred care (Renzaho et al., 2013). Understanding the client's culture and background and establishing proper communication with them contribute to nurses' competence in client‐centredness (Eriksson et al., 2015).

The nurses in this study reported fair level of CCC, which is similar to other study findings highlighting nurses' moderate competence to provide care for culturally diverse clients (Cicolini et al., 2015; Schenk et al., 2022; Wahlström et al., 2020) and the importance of cultural competence, among other competencies (Cicolini et al., 2015; Osmancevic et al., 2023; Wahlström et al., 2020). However, another research reported low levels of cultural competence and cultural knowledge among professionals in health and social services when working with immigrants in European countries (Gil‐Salmerón, 2021). Moreover, among healthcare professionals, nurses tend to have significantly lower level of cultural competence (Casillas et al., 2014; Rakic et al., 2022). It has been suggested to implement effective educational training for nurses to develop their cultural competence and eliminate health disparities (Osmancevic et al., 2023). Regarding the domains of CCC, our result demonstrated that RNs had the least competency in the emotions/empathy domain. In CCC, emotion/empathy refers to feeling and emotional reaction of professionals when dealing with culturally diverse clients and being comfortable and multiculturally empathic (Bernhard et al., 2015). Emotion and motivation are related concepts where emotion could be derived from motivational activity and motivation could be induced by emotional experience (Lazarus, 2006). Emotional states arising from encountering clients from diverse backgrounds could influence a person's comprehension of the client's culture (Hietapakka et al., 2019). Empathy is an important part of cross‐cultural competency (Bernhard et al., 2015; Lorié et al., 2017) which influences healthcare quality (Lorié et al., 2017). In another study conducted on the cultural competence of nurses in Finland, emotions/empathy domain was found associated with Finnish and foreign‐born nurses' perceived time pressure and psychological distress (Wesołowska et al., 2018). More training on the development of cultural competence and emotion/empathy domain among Finnish RNs is recommended.

We found a positive association between ICCs and CCC and its dimensions, including skills, motivation/curiosity and emotions/empathy, which highlighted the importance of CCC in promoting integrated care. Culture is a dynamic concept, and cultural groups constantly change their origins; thus, becoming culturally competent is a continuous process (Alizadeh & Chavan, 2016). Focusing on multicultural approaches alone when dealing with multiculturalism may result in stereotypes rather than better cultural competence (Epner & Baile, 2012). Therefore, the focus should shift from ‘being’ to ‘becoming’ culturally competent and improving client‐centred care in the healthcare setting (Campinha‐Bacote, 2011). Cultural competence has a positive effect on patient‐centred care models aimed at improving immigrants' satisfaction and practitioners' cultural knowledge, awareness and sensitivity (Renzaho et al., 2013). Although the concepts of cultural competence and client‐centred competence overlap and show similarities in considering each individual's uniqueness and improving the quality of care, they both aim to improve various aspects of health quality. While client‐centred care targets the general population to improve its health status by providing personalized care, CCC focuses on ensuring health equity for marginalized groups in society (Beach et al., 2006). The existing literature has suggested that the barriers to providing high‐quality care for immigrants include the following: insufficient language skills; different views on health and disease; mismatched expectations; poor adherence to treatment, which might result in prolonged consultation sessions; and experiences of discrimination (Clough et al., 2013; Filler et al., 2020), health disparities (Northridge et al., 2020; Schneider et al., 2015) and inequality (Schneider et al., 2015).

Nearly half of our participants had received multicultural education in their degree, which was similar to the other study findings conducted by Shepherd et al. (2019). However, most of them expressed a lack of additional multicultural training (e.g. at the workplace), which echoed former studies by which healthcare professionals reported receiving insufficient cultural training (Antón‐Solanas et al., 2022; Filler et al., 2020). Moreover, our findings showed a positive association between cultural training and ICCs which supports the role of cultural education in development of ICCs and providing client‐centred care for immigrants. Likewise, in the study of Asempapa et al. (2021) higher self‐reported competencies in integrated care were found associated with integrated care training, including cultural competence training, and working experience. Other studies have confirmed that education on multiculturalism and exposure to multicultural environments are associated with higher cultural competence (Cicolini et al., 2015; Dobrowolska et al., 2020; Osmancevic et al., 2023; Schenk et al., 2022; Wahlström et al., 2020), and they have highlighted the importance of cross‐cultural education in healthcare professionals' competence development (Paric et al., 2021; Shepherd et al., 2019). Despite the integration of cultural competence in Finnish nursing education, the level of implementation of cultural competence in nursing curricula and its evaluation varies among nursing programmes, and systematic work on integrating and evaluating cultural competence in Finnish nursing education is required (Paric et al., 2021). Moreover, providing a multicultural training framework for nurses working in integrated healthcare is recommended, as developed countries are more affected by immigration growth (Trost et al., 2018). In this regard, the integration of cultural education in European healthcare education is emphasized (Sairanen et al., 2013), and European healthcare systems should address the needs of the marginalized population of immigrants by providing culturally tailored healthcare services and respecting their culture (Devillé et al., 2011).

Among other background variables influencing ICCs in this study, older nurses with more than 1 year of work experience in a private health setting, having a higher competence for working in a multicultural environment, and receiving additional multicultural training had a positive association with higher ICCs. Likewise, an earlier study showed that working experience, age, education and educational programmes are positively associated with higher nurse competence (Flinkman et al., 2016). Public health nurses and midwives in Finland are involved in health promotion services, including maternity and child welfare clinics, school and student healthcare, and home care, playing a fundamental role in providing preventive care and early interventions in primary healthcare in the public sector. Hence, more training for the development of ICCs and CCC for novice RNs and those working in public healthcare is recommended.

### 5.1 Strengths and limitations of the study

This study evaluated the ICCs and CCC of Finnish RNs with a random sampling method and presented their association prior to the national reformation in the country's social and healthcare. Despite its contributions, it has several limitations that should be acknowledged. The study participants self‐evaluated their competencies, which might have biased the findings due to desirability and overestimation effects. The cross‐sectional study design could not determine a causal relationship between ICCs and CCC. The low response rate to the online survey limited the generalizability of the findings to the entire study population. However, the sample size was sufficient for the detection of an association between ICCs and CCC using a linear regression model based on a coefficient of 0.30, power of 90%, alpha of 5%, SD of 0.5 for ICCs and 0.3 for CCC. The risk of auto‐generated responses by malware could have been addressed in the questionnaire design or during data collection. Protective measures should be considered in the future studies using online survey to minimize the potential risk. The majority of participants in our study were women RNs, more research is recommended on the ICCs of various social and healthcare workforce groups from a variety of gender identities. Although we used a validated tool for measuring the CCC of RNs and the internal consistency of items on each domain was in the acceptable range, the total Cronbach's alpha for all variables in the CCC tool was low (0.42), which might have been due to the low number of items in the tool. The immigration rate is low but growing in Finland, and Finnish public healthcare is required by the constitution to provide equal social and health services to all residents. Hence, the application of this study result may be limited to RNs working in countries with similar immigration rates and healthcare structures.

### 5.2 Recommendations for further research

Further studies on the competencies of health and social care professionals are recommended to evaluate the ICCs and CCC of professionals in the integrated care services working at different units (i.e. acute care, intensive care unit, social service) and cities (with small and large numbers of immigrants) and providing various levels of care (primary, secondary and tertiary).

## 6 CONCLUSION

The transition of social and healthcare to providing integrated care demands that professionals have competencies such as client orientation, enabling clients to make health‐related decisions for themselves and close relatives, providing accessibility to different services and clarity of services. The high level of ICCs of RNs in this study indicated their ability to coordinate and deliver care. The association found between ICCs and CCC highlighted the importance of ICCs in relation with CCC and underlined the importance of promoting both ICCs and CCC in nursing practice and education. It is recommended that public healthcare organizations implement training on the development of CCC for RNs working with clients from diverse backgrounds and that nurse managers support novice RNs in the public health sector through in‐service training for the development of ICCs. Nurse educators should foster the integration of multicultural education in the nursing curricula to enhance the cultural competence of RNs, as integrated and people‐centred care is not possible without considering the cultural needs of immigrants and their communities. With the increasing immigration rate in recent years in Europe, the cultural competence of social care and healthcare professionals should be under the spotlight in health projects and services, especially when providing integrated and people‐centred care to immigrants.

## AUTHOR CONTRIBUTIONS

**Maliheh Nekouei Marvi Langari** provided the conceptualization, methodology, formal analysis, funding acquisition, writing the original draft preparation, review and editing. **Tarja Heponiemi** provided the conceptualization, methodology, formal analysis, funding acquisition, review and editing. **Jaana Lindström, Anu‐Marja Kaihlanen, Laura Hietapakka, Hamid Heidarian Miri and Hannele Turunen** provided the conceptualization, methodology, formal analysis, review and editing.

## FUNDING INFORMATION

The study was supported by the Strategic Research Council of the Academy of Finland (Project 303607) and the Antti and Jenny Wihuri Foundation (2018/00180257). However, we confirm that the funders were not involved in designing the study, collecting and analysing data and reporting the findings.

## CONFLICT OF INTEREST STATEMENT

The authors declare no conflict of interest.

## ETHICS STATEMENT

The completion of the survey was considered to indicate consent. We ensured voluntary participation and secured the participants' anonymity using the self‐administered online questionnaire sent via email and allowing the union to distribute the questionnaire among its members rather than the research group. Data were stored securely, and only members of the research team could access these data to store and process them. Ethical approval for this study was granted by the Finnish Institute for Health and Welfare Ethics Board (THL/253/6.02.01/2018) under the ethical guidelines for research involving human subjects established by the Finnish National Board on Research Integrity TENK (2019).

## Data Availability

The dataset used in the current study is available from Tarja Heponiemi (tarja.heponiemi@thl.fi) upon reasonable request.
